# Urine-Derived Epithelial Cell Lines: A New Tool to Model Fragile X Syndrome (FXS)

**DOI:** 10.3390/cells9102240

**Published:** 2020-10-05

**Authors:** Marwa Zafarullah, Mittal Jasoliya, Flora Tassone

**Affiliations:** 1 Department of Biochemistry and Molecular Medicine, School of Medicine, University of California Davis, Sacramento, 95817 CA, USA; mzafarullah@ucdavis.edu (M.Z.); mjjasoliya@ucdavis.edu (M.J.); 2 MIND Institute, University of California Davis Medical Center, Sacramento, 95817 CA, USA

**Keywords:** Fragile X syndrome, epithelial cells, *FMR1 mRNA*, FMRP, neurodevelopmental disorders, urine-derived cells

## Abstract

Fragile X syndrome (FXS) is an X-linked neurodevelopmental condition associated with intellectual disability and behavioral problems due to the lack of the Fragile X mental retardation protein (FMRP), which plays a crucial role in synaptic plasticity and memory. A desirable in vitro cell model to study FXS would be one that can be generated by simple isolation and culture method from a collection of a non-invasive donor specimen. Currently, the various donor-specific cells can be isolated mainly from peripheral blood and skin biopsy. However, they are somewhat invasive methods for establishing cell lines from the primary subject material. In this study, we characterized a cost-effective and straightforward method to derive epithelial cell lines from urine samples collected from participants with FXS and healthy controls (TD). The urine-derived cells expressed epithelial cell surface markers via fluorescence-activated cell sorting (FACS). We observed inter, and the intra-tissue CGG mosaicism in the PBMCs and the urine-derived cells from participants with FXS potentially related to the observed variations in the phenotypic and clinical presentation FXS. We characterized these urine-derived epithelial cells for *FMR1* mRNA and FMRP expression and observed some expression in the lines derived from full mutation mosaic participants. Further, FMRP expression was localized in the cytoplasm of the urine-derived epithelial cells of healthy controls. Deficient FMRP expression was also observed in mosaic males, while, as expected, no expression was observed in cells derived from participants with a hypermethylated full mutation.

## 1. Introduction

Neurodevelopmental disorders are conditions categorized by impairments of intelligence or social skills, with an onset in the developmental period. Fragile X syndrome (FXS), the most common inherited cause of intellectual disability (ID) and the single leading monogenic currently known cause of Autism Spectrum Disorder (ASD) [1], is caused by an expansion of a CGG trinucleotide repeat, greater than 200, in the 5’ untranslated region of the fragile X mental retardation1 (*FMR1*) gene. This leads to transcriptional silencing of the gene with consequent absence of the encoded product, the fragile X mental retardation protein (FMRP), which plays a crucial role in synaptic plasticity and memory. Males with FXS have mild to severe ID, and while 70% of the girls are less affected, they typically present with learning problems. Behavior problems include anxiety, sensory hyperarousal, hyperactivity, social deficits, language deficit, and obesity.

Size and methylation mosaicism of the CGG repeats has been observed in many patients with FXS. Pretto and colleagues [2] suggested that in individuals with FXS, mosaicism (size or methylation) can result in low FMRP expression, which may be enough to impact their cognitive functions positively. Early studies reported that 38% of patients with FXS were mosaics, presenting with both premutation and full mutation alleles [3]. Further, a small screening study found 80% of mosaicism among the identified individuals with FXS, suggesting that mosaicism is quite common in FXS [4].

Due to the high prevalence of neurodevelopmental disorders, including FXS, desirable cells for investigation and cell therapy would be ones that can be generated by a simple isolation and culture method using a donor sample obtained in a non-invasive manner. To date, the collection of different donor-specific cells that can be isolated from peripheral blood, skin, and hair require invasive methods for sample isolation and incorporate complicated, costly reagents, time-consuming for the culturing process. These cells also take a considerable time for their in vitro isolation and expansion. Previous studies have suggested that donor-derived urine cells offer a cost-effective and straightforward method of isolating cell lines. Moreover, urine-derived primary cells are a source for generating induced pluripotent stem cells (iPSCs) and have been applied as modeling platforms for various disorders, including kidney disorders, muscular dystrophy, and paroxysmal kinesigenic dyskinesia [5,6,7,8]. Importantly, iPSCs derived from urine are shown to possess the differentiation potential into functional glutaminergic, dopaminergic, and motor neurons [8,9], whose impairment has been well documented in FXS.

This study reports, for the first time, an optimized protocol for establishing FXS patient-primary cell models derived from urine samples. We determined the percentage of epithelial, hematopoietic, and erythroid cells in the established cell population using flow cytometry. Additionally, we show that FXS-derived epithelial cells had 0.1% residual FMRP compared to healthy-individual derived epithelial cells by two independent methods. Because the loss of FMRP is the primary cause of FXS, our data suggest that FXS patient’s urine-derived epithelial cells are a viable option in understanding the disease’s underlying molecular mechanisms, potentially leading to therapeutic intervention.

Overall, FXS patient urine-derived epithelial cells can be a less invasive primary cell model for understanding the molecular mechanisms of FXS pathogenesis. It can also be used as personalized modeling of an individual’s response to various drugs and identifying potentially effective therapy based on their genetic makeup.

## 2. Materials and Methods

### 2.1. Study Participants

As part of an ongoing metformin clinical trial at the UC Davis MIND Institute, urine samples were collected from 25 participants with FXS, including 24 males and one female, age (6–25 years) and seven healthy individuals (TD), including two males and five females, age (16–32 years). Participants of this study or their parents/legal guardians signed a written informed consent to provide urine samples for epithelial cell generation and further experimentation following a protocol following the Institutional Review Board at the University of California, Davis. Nineteen epithelial cell lines derived from 12 male participants with FXS and seven TD (two males and five females) were established (Table 1).

### 2.2. Collection of Urine

For the best results, participants were advised to drink a full glass of water about one hour before collecting, and urine samples were processed within 1–2 h. Urine samples were collected into a sterile container after cleaning the urethral area with disinfectant wipes immediately before collection. To significantly reduce the opportunities for contaminants to enter the urine stream during the collection of the specimen, the first portion of the urine stream was discarded, and 30–50 mL was then collected into a clean container. Urine samples were placed on ice or at 4 °C immediately after collection to avoid a decreased yield due to prolonged processing time.

### 2.3. Primary Culture of Urine-Derived Epithelial Cells

Twelve well plates were coated with iMatrix (Reprocell, Beltsville, MD, USA) before processing the urine sample. For coating, 4.8 uL iMatrix-511 solution was added to 1 mL Dulbecco’s phosphate-buffered saline (DPBS) (Thermo Fisher Scientific, Waltham, MA, USA) per well, followed by mixing and incubating at 37 °C for 3 hr. or at 4 °C overnight. iMatrix solution was aspirated immediately before plating the cells to avoid dryness of the coating solution. For processing, midstream urine was collected, followed by transferring it into a sterile 50 mL centrifuge tube. Samples were centrifuged at 1000 rpm for 10 min at room temperature. The supernatant was carefully aspirated, leaving only 1–2 mL into the tube above the cell pellet. Cell pellets were then washed once with 10 mL of washing buffer (500 mL DPBS supplemented with 50 mg ml^−1^ Primocin (Invitrogen, San Diego, CA, USA) and resuspended into 2 mL of primary media. Cells were plated into iMatrix coated well plates and incubated at 37 °C for 24 h. Primary media consisted of DMEM/F12 + GlutaMAX nutrient mix (1:1) (Thermo Fisher Scientific, Waltham, MA, USA), supplemented with 10% (*vol*/*vol*) human serum (Sigma-Aldrich, St Louis, MO, USA), 50 mg mL^−1^ of Primocin (Invitrogen, San Diego, CA, USA) and, the REGM SingleQuot kit supplements (Lonza, Basel, Switzerland) (do not use FBS to reduce background). The media was filter-sterilized using a 0.2 µm filter unit and then supplemented with 50 mL human serum. On day two, media was removed, and 1 mL of primary fresh medium was added to the plated cells. Cells were fed until small colonies of approximately 5–6 dense cells appeared, which took 2–3 weeks after plating and reaching 80–90% density after 24 days of plating.

### 2.4. Proliferation of Urine-Derived Epithelial Cells

Cells were harvested with TrypLE^™^ (Thermo Fisher Scientific, Waltham, MA, USA), counted using a hemocytometer, and viability was determined by trypan blue. They were resuspended into complete primary culture media and then plated into iMatrix coated six wells plate (or directly transfer into the coated T-25 flask, depending on cell density) and incubated at 37 °C. Media was changed every other day until 70–80% confluency was reached (~10^6^ cells), mostly after Day 6 of plating into T-25. After harvesting the cells at passage 1 (P1), cells were split 1:4 into T-25 plates for further expansion and aliquoted 3:4 into 1 mL of CryoStem^™^ Freezing Medium (Thermo Fisher Scientific, Waltham, MA, USA) in cryotubes and aliquots were stored in liquid nitrogen.

### 2.5. Fluorescence-Activated Cell Sorting (FACS)

Aliquots from cultured urine-derived cells from P1 were thawed in RPMI supplemented with 10% FBS, and 1 uM DNase I. Cells were washed with PBS, stained with RayBright LIVE 780, and washed with FACS buffer. Cells were blocked with FcR blocking buffer and surface stained with antibody CD45 (RayBright 488), CD235a (BV421), CD326 (PE). After staining, cells were washed with a FACS buffer, fixed and permeabilized with RayBio fixation and permeabilization buffer for 20 min. Finally, cells were stained with Alexa Fluor 647 anti-Cytokeratin 14 in a permeabilization buffer for 30 min. After staining, the cells were analyzed using a fluorescence-activated cell sorter (RayBiotech, GA, USA).

### 2.6. CGG Repeat Allele Size and Methylation Status

Genomic DNA (gDNA) was isolated from 1 × 10^6^ derived epithelial cells and from 3 mL of peripheral blood using the Gentra Puregene Blood Kit (Qiagen, Valencia, CA, USA). CGG repeat allele size and methylation status were assessed using a combination of PCR and Southern blot analysis on DNA isolated from peripheral blood and by PCR on DNA isolated from the epithelial cells. PCR was carried out using *FMR1* specific primers (AmplideX PCR/CE, Asuragen, Inc.), and amplicons were visualized by capillary electrophoresis and analyzed as previously reported [10]. Southern blot was performed using the Stb12.3 *FMR1* specific chemiluminescent intronic probe, as detailed in [11].

### 2.7. mRNA Expression Levels

Total RNA was isolated from 1 × 10^6^ urine-derived epithelial cells using Trizol (Thermo Fisher Scientific, Waltham, MA, USA) and quantified using the Agilent 2100 Bioanalyzer system. RNA isolation was performed in a clean and RNA designated area. cDNA was synthesized, as previously described [12]. *FMR1* transcript levels and of the reference gene β-glucuronidase (*GUS*) were measured by qRT-PCR using either Assays-On-Demand from Applied Biosystems (Applied Biosystems, Foster City, CA, USA) or custom-designed TaqMan primers and probe assays as previously described [12].

### 2.8. Western Blot Analysis

Cells were lysed using lysis buffers (Cell Signaling Technology, Inc., Danvers, MA, USA) supplemented with a complete protease inhibitor cocktail (Roche Applied Science, Penzberg, Germany) and phenylmethylsulfonyl fluoride (Sigma-Aldrich Corp, St Louis, MO, USA). Lysates were centrifuged at 1,6000 rpm for 15 min at 4 °C to remove cellular debris followed by protein quantification using Bradford assay (BioRad Laboratories, Inc. Hercules, CA, USA). Ten µg protein was loaded on a 4–12% Bis-Tris gels (BioRad Laboratories, Inc. Hercules, CA, USA) and run at 80 V for 30 min and 110 V for 90 min. Resolved proteins were then transferred onto nitrocellulose membranes using the Trans-Blot Turbo transfer system (BioRad Laboratories, Inc. Hercules, CA, USA) at 25 V, 1.0 A 30 min. Membranes were stained with Ponceau to test for transfer efficiency, blocked with 3% milk for 1 hr at room temperature followed by incubation with 1:1000 diluted FMRP primary antibodies (MAB 2160, MiliporeSigma, Burlington, MA, USA) overnight at 4 °C. Membranes were then washed in 1X-TBST and incubated with HRP linked secondary antibody diluted 1:1,0000 (Catalog# 1706516, Biorad Laboratories, Inc. Hercules, CA, USA) for 1 hr at room temperature. Bands were then visualized using Chemiluminescent substrate, Super Signal West Dura (Thermo Fisher Scientific, Waltham, MA, USA). Densitometry analysis of bands for relative protein quantification was performed using the Alpha Innotech Gel Imaging System (Cambridge Scientific, Watertown, MA).

### 2.9. Immunofluorescence Staining

Urine-derived epithelial cells were grown in primary media on 22 mm × 22 mm iMatrix coated glass coverslip for 24 h at 37 °C. Cells were fixed for 10 min at room temperature in DPBS containing 4% paraformaldehyde, washed in PBSt (DPBS containing 10% Tween 20), permeabilized in 0.1% Triton X-100 in DPBS at Room temperature for 10 min and washed three times in PBSt for 5 min each. Permeabilized cells were blocked with 5% goat serum for 1 hour at room temperature, then incubated overnight at 4 °C with purified anti-FMRP antibody (5 ug/mL) (BioLegend, San Diego, CA, USA) in diluted 5% goat serum and washed three times in PBSt for 5 min each. Cells were incubated in Alexa-488-coupled anti-mouse immunoglobulin antibody (2 ug/mL) (Thermo Fisher Scientific, Waltham, MA, USA) in 5% goat serum at room temperature for one h and then washed three times in PBSt for 5 min each. Once washed, cells were incubated in DAPI (4’,6-Diamidino-2-Phenylindole, Dihydrochloride) by (Thermo Fisher Scientific, Waltham, MA, USA) at room temperature for 5 min. After washing with PBSt at room temperature and mounted in Vectashield mounting media (Vector Laboratories, Burlingame, CA, USA), cells were visualized using an Olympus FLUOVIEW FV1000 confocal microscope. Pictures of different samples were blindly taken using the same settings.

## 3. Results

### 3.1. Isolation and Expansion of Urine-Derived Epithelial Cells

**Figure 1:**
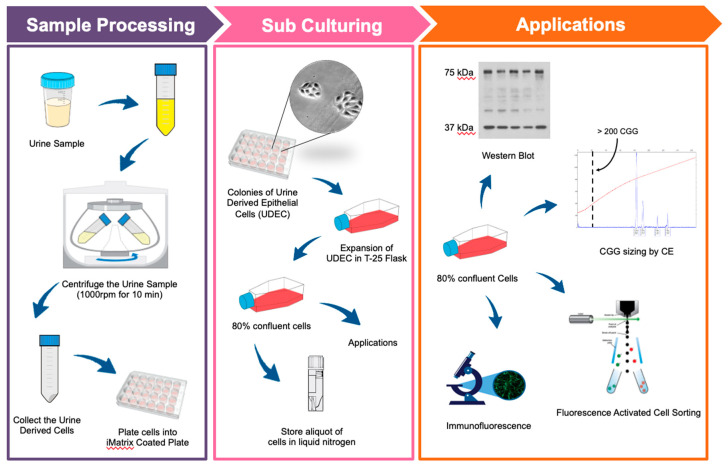
Schematic representation of the urine-derived epithelial cell culture establishment protocol. For sample processing, a urine sample was collected, transferred into a sterile 50-mL centrifuge tube, and centrifuged at 1000 rpm for 10 min at room temperature. Collected urine-derived cells were plated into iMatrix coated wells and incubated at 37 °C for 24 h. Cells were fed until small colonies of approximately 5–6 cells appeared, which on average, took 2–3 weeks after plating to reach 80–90% density. Cells were harvested, aliquoted, and stored in cryotubes in liquid nitrogen for different applications (Western Blots, CGG repeat sizing, Immunofluorescence, and Fluorescence-Activated Cell Sorting).

**Figure 2:**
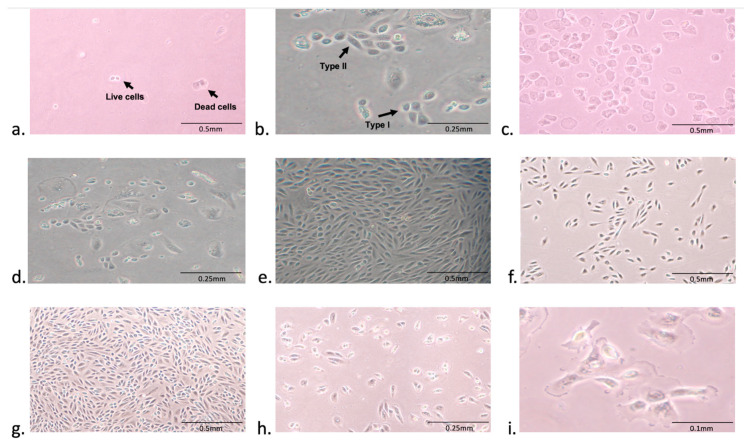
Isolation and expansion of urine-derived epithelial cells. (**a**) A representation of living and dead cells in urine samples. (**b**) Urinary cell morphologies: Type I shows a smooth-edged contour, and Type II, have cobblestone-like cell morphology with random arrangements. (**c**) Squamous cells in the urine sample of female individuals. (**d**) Epithelial cell colonies appear within 2–3 weeks after plating. (**e**) 70–80% cell confluency. (**f**) Expansion of epithelial cells at P1 in the T-2 flask. (**g**) 80% cell confluency in a T-25 flask. (**h**) The number of cells decreased after four passages. (**i**) The slow growth of epithelial cells at later passages. 10× magnification was used for image (**a**,**c**,**e**–**g**) while (**b**,**d**,**h**) have been captured at 20× magnification, the 40× magnification was used for the image (**i**).

A total of 32 urine samples were collected from 25 FXS and 7 TD individuals by following a protocol, as illustrated in (Figure 1), under a sterilized environment. No bacterial contamination was present in any of the cultured samples. The average number of live cells in these samples was 4–10, as measured by trypan blue staining. Living cells were attached to the coated plate and started expanding within 2–3 weeks, while the dead cells didn’t attach and were removed from the culture media (Figure 2a). Consistently with previous reports, we observed both types of urinary cell morphologies: type I, that showed a smooth-edged contour and, type II, that had a cobblestone-like cell morphology with random arrangements [13,14]. Interestingly, we found that both types of colonies were present in the same specimen, although type II usually became more prevalent (Figure 2b). Besides, we found that urine from females mainly consisted of squamous cells (Figure 2c). After plating the cells in primary media, it took about 2–3 weeks for the colonies to appear (Figure 2d). Once cells began growing, they expanded rapidly, becoming 70–80% confluent in 6–7 days (Figure 2e). Once harvested at passage 1 (P1) and transferred (~0.2*10^6^ cells) to a T-25 flask (Figure 2f), cells only took less than a week to reach 80% confluency one × 10^6^ cells) (Figure 2g). However, after four passages, the number of cells in primary culture (Figure 2h) did not reach a cell density of more than 25% (Figure 2i).

### 3.2. Urine-Derived Cells Expressed Epithelial Cell Surface Markers

**Figure 3:**
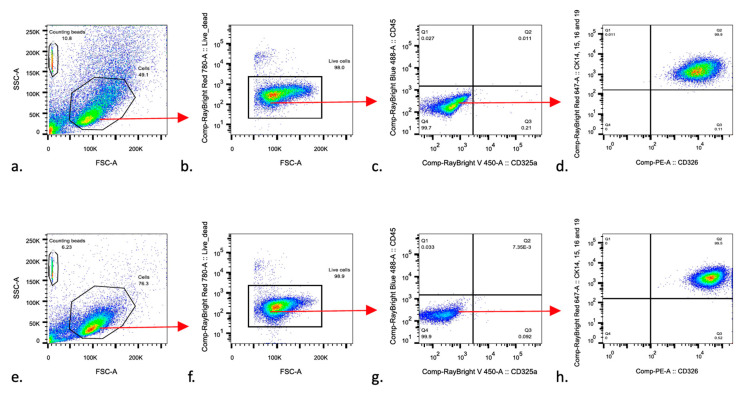
Urine-derived cells express epithelial cell surface markers. Fluorescence-activated cell sorting analysis of urine-derived epithelial cells at P1 from Case 1 (top panel, **a**–**d**) and Case 3 (bottom panel, **e**–**h**). Cells were stained with antibodies against epithelial surface markers pan-cytokeratin (CK14, CK15, CK16, CK19) and CD326. In addition, cells were stained with antibodies to general hematopoietic cell marker CD45 (RayBright 488) and a marker for the human erythroid cells and their progenitors CD235a (BV421). From left to right, the cells were separated from precipitation and debris by gating them for further analyses (as shown by the red arrow). Only alive cells (**b**,**f** corresponding to 98% and 98.8% of alive cells, respectively) were included in the analysis. The cells stained negative for CD45 and CD325a (**c**,**g**) while stained strongly positive for the CD326, CK14, CK15, CK16, and CK19 (**d**,**h**).

To validate that the newly generated urinary cell colonies were epithelial cells and distinguish them from other urinary cell types, we performed Fluorescence-Activated Cell Sorting (FASC) using a marker of human epithelial cells, white and red blood cells (Figure 3). FACS revealed that Urine-derived Cells at P1 stained 48–76% positive for surface markers characteristic of epithelial cells, i.e., pan-cytokeratin (CK14, 15, 16, 19) and CD326. Besides, these cells stained 0.013–0.025% for the general hematopoietic cell marker CD45 and 0.07–0.1% for the CD235a, a marker for the human erythroid cells and their progenitors (Supplementary Material Table S1), indicating that the established cell lines were mostly constituted by epithelial cells and not by hematopoietic or erythroid progenitor cells.

### 3.3. Intra- and Inter-Tissue Mosaicism Detected in PBMCs and Urine-Derived Epithelial Cells

**Figure 4:**
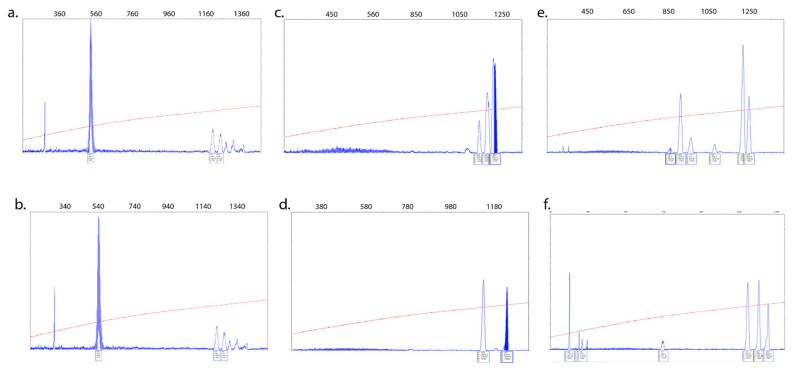
Size mosaicism occurs between PBMCs and urine-derived epithelial cells. Representative capillary electropherograms of three individuals with a full mutation are illustrated. Several similar peaks, each representing single distinct alleles, were observed with the similarity between PBMCs (**a**) and epithelial cells (**b**) [Case 11]. Interestingly, a different CGG profile between PBMCs (**c**,**e**) and epithelial cells (**d**,**f**) [Case 7 and Case 2 respectively] and within the two tissues was observed in two other cases indicating the presence of both inter and intra-tissue mosaicisms. The X-axis marks the size of the alleles in base pairs. The *Y*-axis marks the fluorescence intensity of each allele. The numbers inside the boxes indicate H: Height, A: Area, S: Size, D: Datapoint. Only the S number (base pairs) is used to calculate the CGG repeat number.

CGG repeat size of the *FMR1* allele was determined in both peripheral blood mononuclear cells (PBMCs) and urine-derived epithelial cell samples from participants (n = 10). Interestingly, we observed no difference in the CGG repeat pattern between PBMCs (Figure 4a) and the urine-derived epithelial cells (Figure 4b) from the same individual. However, we did observe significant differences between PBMCs (Figure 4c,e) and urine-derived epithelial cells and the CGG allele distribution in other cases (Figure 4d,f), suggesting the presence of inter-tissue mosaicism. In addition to inter-tissue differences between PBMCs and urine-derived epithelial cells, we also observed, in some cases, multiple CGG size alleles within the same tissues (Figure 4a,c,e) representing intra-tissue mosaicism.

### 3.4. Urine-Derived Epithelial Cells Express FMR1 mRNA and FMRP Protein

**Figure 5:**
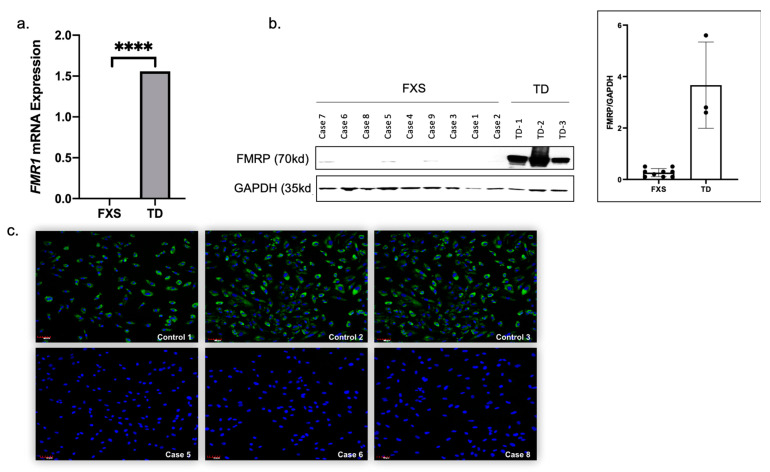
Urine-derived epithelial cells expressed *FMR1* mRNA and FMRP protein. (**a**) Bar plot showing the significantly higher expression (*p*< 0.0001) of *FMR1* mRNA in TD (n = 1) as compared to FXS patients (n = 5). (**b**) Western blot protein expression patterns showing complete loss or significantly lower (<= 0.1%) FMRP expression (n = 9, *p* < 0.0001) in FXS patient derived epithelial cells compared to TD (n = 3). Data also show the ratio of FMRP protein/GAPDH. (**c**) Confocal images and *in-situ* immunofluorescence showing high expression of FMRP protein (green) localized to the cytoplasm of the epithelial cells derived from normal individuals (Top panel; n = 3), while complete loss or low FMRP expression has been observed in the cells derived from FXS patients (Bottom panel; n = 3).

Expression of the *FMR1* mRNA and FMRP was measured in a subgroup of the established epithelial cells derived from participants with FXS and TD. The *FMR1mRNA* expression levels, normalized against the GUS gene, were, as expected, significantly higher (*p* < 0.0001) in TD (n = 1) as compared to FXS participants (n = 5) (Figure 5a). FMRP expression was measured using Western blot analysis. We observed a complete loss or significantly lower (<=0.1%) FMRP expression (n = 9, *p* < 0.0001) in patients with FXS derived epithelial cells compared to TD (n = 3). Interestingly, we observed a minimal amount of FMRP expression by Western blot analysis in protein extracts derived from patients with mosaicism, including Case 5, Case 7, and Case 9, but only after a long exposure time. We further confirmed FMRP expression and its localization in epithelial cells using in-situ immunofluorescence. Consistently with Western blot analysis, high FMRP expression, localized in the cytoplasm of the epithelial cells derived from TD, was detected. In contrast, complete loss or low FMRP expression was observed in the cells derived from FXS participants with a fully methylated full mutation (Table 1, Case 5, Case 6, and Case 8) (Figure 5c). Although Case 8 was present with 85% methylation, we did not detect any FMRP expression by immunofluorescence or Western blot analysis, likely due to a deficit in translational efficiency of the large unmethylated alleles (240–350 CGG repeats; see Table 1).

### 3.5. Factors Affecting the Establishment of Urine-Derived Epithelial Cells

**Figure 6:**
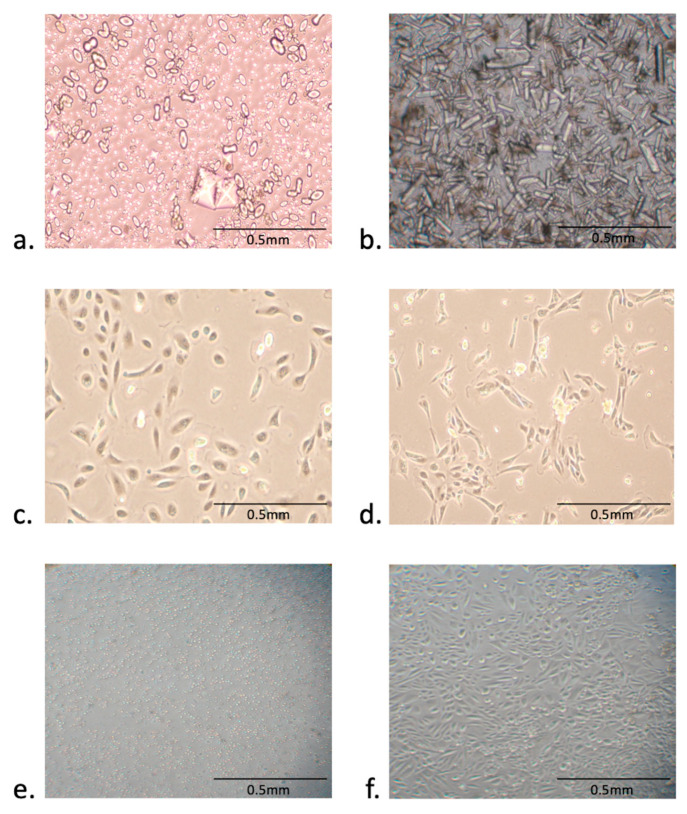
Factors affected urine-derived epithelial cells (**a**,**b**). Representation of high debris in the urine samples of FXS patients. (**c**,**d**) Plate coating material impacts the growth and morphology of epithelial cells. Normal growth on iMatrix coated plates (**c**) v/s distorted mesh-like growth pattern of cells on Poly L-lysine (**d**–**f**) Urine storage conditions affect the growth of the derived epithelial cells. No cell growth was observed from the urine samples stored for 24 h at −20 °C before isolating cells (**e**) as compared to freshly collected and processed urine samples (**f**).

In our experience, we observed that the health status of the donor affected the urine-derived cells attachment and growth in vitro, as the large debris in the urine samples decreases the growth of the healthy cells (Figure 6a,b). To find if the plate coating material had an impact on the growth of the epithelial cells, we compared the widely used coating material Poly L-lysine with iMatrix. Interestingly, we also found that the plate coating material impacts the growth and morphology of epithelial cells as we observed healthy growth on iMatrix coated plates (Figure 6c) while cell morphology and health appeared to be compromised as we observed distorted mesh-like pattern when cells were grown on plated coated with Poly L-Lysine (Figure 6d). To determine whether the urine sample could be stored for transportation before culturing the epithelial cells, we froze the portion of freshly donated urine for 24 hr at −20 °C before isolating and culturing the cells by our standard procedure. Significantly, on Day 12, we didn’t observe any cell growth from the urine samples stored for 24 h at −20 °C (Figure 6e) as compared with cells isolated from freshly collected urine samples (Figure 6f).

## 4. Discussion

Urine-derived epithelial cells present a unique cell source with proliferation ability and an advantageous in vitro model to study FXS. They could be used to investigate FXS disease mechanisms, identify new biomarkers, evaluate therapeutic approaches, generating iPSCs, and be used for drug screening. As urine can be collected by totally non-invasive procedures, this method can be used universally for any neurodevelopmental disease. Using this non-invasive approach, we have generated 19 epithelial cell lines, and we are continuing to create more lines to be used for future basic and translational studies in FXS. To the authors’ knowledge, this paper is the first report on establishing epithelial cell lines from the urine of the participants with FXS and proposed as an in vitro model to investigate the mechanism of disease development.

One of our initial concerns was determining whether the urine-derived cells from individuals with FXS could expand in vitro as efficiently as other cell types. We observed that, in our hands, cell colonies arising within 2–3 weeks of isolation could be established as a stable cell population within just the following six days. The second biggest concern was to make sure that the established cell lines were constituted by epithelial cells. FACS analysis determined that the majority of cells harvested at P1 were indeed epithelial cells as defined by the presence of epithelial-specific surface markers, i.e., pan-cytokeratin (CK14, 15, 16, 19) and CD326. Moreover, we detected the expression of *FMR1* mRNA and FMRP in these cells, although, as expected, we observed low expression levels in the cells derived from participants with FXS compared to TD.

CGG size mosaicism is common in individuals with the *FMR1* full mutation [2,15,16,17,18,19]. Although, depending on the allele size and the methylation status, FMRP can be produced, mosaic individuals usually present with developmental delay due to the low *FMR1* gene expression and the inefficient translation of the extended CGG repeat mRNA. In addition, mostly, DNA testing is performed on PBMCs, which results may not accurately show the mutation pattern in other tissues, such as the brain. Mosaicism in different tissues has been investigated and reported [2,17,20,21,22], with similarities across tissues in some cases and extreme differences in others. Thus, it is difficult to predict on an individual basis whether the *FMR1* mutation observed in blood will show the same or different pattern in other tissues. For this reason, we assessed the impact of CGG size mosaicism in the participants by comparing DNA isolated from both PBMCs and urine-derived cells. Consistent with other reports, we observed mosaicism in some cases, with a similar CGG allele size and distribution between the two different tissues, while different in others, underling the complexity of the CGG repeat instability. The observed intra-and inter-tissue mosaicism, as demonstrated by the presence of multiple size alleles and methylation status in PBMC’s and across different tissues, could be associated with the variability in the wide spectrum of clinical involvement in FXS.

The difficulties presented in obtaining tissues from patients with neurodevelopmental disorders and lack of adequate preclinical models with high predictive and translational power pose limitations in the study of these disorders and in developing effective target treatments. As brain biopsies are impractical and risky, many studies develop methods to differentiate urine-derived cells into neural-lineage cells [23,24,25]. The human urinary cells represent a promising source of stem cells as they can also be converted into neural stem cells by using a non-integration-free method with small molecules, which is less time consuming than going through iPSCs [26]. As urine-derived stem cells have a similar phenotype to mesenchymal stromal cells (MSC), they can be reprogrammed into iPSCs and converted into astrocytes, oligodendrocytes, and neurons. Thus, urine-derived cells represent an alternative source of cells for developing iPSCs, with the advantage of being collected by a non-invasive method [27]. They may play an essential role in identifying and developing safe and effective therapies for patients with neurodevelopmental conditions, such as FXS. Moreover, this unique epithelial cell modeling and their further differentiation into neural lineage cells will provide valuable information for predicting drug response and assessing environmental disease triggers. Furthermore, the development of epithelial cells provides a new platform in FXS modeling and works in complementary ways; it is expected to benefit research and clinical applications in personalized medicine.

Because of the simplicity, safeness, the low-cost, and the non-invasive process described here, epithelial cell lines can be generated in a relatively short time from a significant proportion of patients with FXS or other neurodevelopmental disabilities, including Autism Spectrum Disorders. Importantly, the establishment of these cell lines, coupled with extensive phenotypic information, including a clinical history of FXS-related comorbidities, will potentially result in a reasonable biobank of cell lines from phenotypically well-characterized individuals with FXS that can be used initially to identify potential molecular biomarkers predictors of drug efficacy, in pharmacology and toxicology tests and then should eventually evolve into a community resource for various advance studies on the pathology of FXS.

One limitation of this study is related to the few numbers of cells and to the amount of the debris and contaminants in the urine sample that significantly decrease the chances of epithelial cell line establishment. Also, it is challenging to collect the first urine and, in enough volume, (~40 mL) from young patients with FXS to ensure the success of the procedure. Thus, optimization and standardization of the urine samples collection method is required to improve the growth and proliferation of urine-derived epithelial cells significantly.

## 5. Conclusions

In summary, there are several advantages in using urine-derived epithelial cells as a tool for FXS modeling: (a) specimens and cells can be easily harvested; (b) epithelial cells do not require processing by enzyme digestion or culture on a layer of feeder cells to support cell growth; (c) since invasive surgical biopsy procedures are not necessary to harvest cells from urine, potential complications, such as urethral or bladder trauma and urinary tract infection and patient morbidity, are avoided; (d) epithelial cells are not exposed to ultraviolet (UV) rays (such as mostly skin fibroblasts or hair follicles do) and thus, they are less likely to contain potential genetic mutations and UV-induced DNA damage; (e) as epithelial cells are autologous somatic cells, there are no ethical issues involved in their use for future preclinical and clinical studies.

## Figures and Tables

**Table 1:** Demographic and molecular information for participants included in the study.

Participants	Age	Gender	Peripheral Blood Mutation Category	Peripheral Blood CGG Repeat Number *	Peripheral Blood (%) Methylation	Mutation Category	Epithelial Cells CGG Repeat Number	Peripheral Blood *FMR1* mRNA Level (StErr)	Epithelial Cells *FMR1* mRNA Level
Case 1	14	M	Full mutation	>200		Full mutation	>200	0	0
Case 2	20	M	Full mutation	>200		Full mutation	>200	0	
Case 3	8	M	Full mutation	>200		Full mutation	>200	0	
Case 4	25	M	Full mutation	>200		Full mutation	>200	0	
Case 5	13	M	Full mutation	>200		Full mutation	>200	0.01 (0.002)	0
Case 6	20	M	Full mutation	>200		Full mutation	>200	0.009 (0.001)	0
Case 7	18	M	Full mutation, Meth mosaic	>200 (30–200) **	>95%	Full mutation, Meth mosaic	>200 (30–200) **	0.29 (0.03)	
Case 8	8	M	Full mutation, Meth mosaic	>200 (240–350) **	85%	Full mutation	>200***	0.47 (0.01)	0
Case 9	8	M	Full mutation	>200***		Full mutation	>200	0.16 (0.004)	0
Case 10	13	M	Full mutation	>200		Full mutation	>200	0	
Case 11	15	M	Full mutation, Size mosaic	>200 (103) **	96%	Full mutation, Size mosaic	>200 (103) **	0.15 (0.06)	
Case 12	17	M	Full mutation	>200		Full mutation	>200	0	

* CGG repeat number measured in peripheral blood. ** Numbers between parenthesis indicate the range of CGG repeat number of unmethylated alleles. *** Very light smear of unmethylated alleles was detected by Southern Blot.

## Supplementary Materials

The following are available online at https://www.mdpi.com/2073-4409/9/10/2240/s1, Table S1. FACS analysis of Urine-Derived Cells.

## References

[1] Harris J.C. (2014). New classification for neurodevelopmental disorders in DSM-5. Curr. Opin. Psychiatry.

[2] Pretto D., Yrigollen C.M., Tang H.-T., Williamson J., Espinal G., Iwahashi C.K., Durbin-Johnson B., Hagerman R.J., Hagerman P.J., Tassone F. (2014). Clinical and molecular implications of mosaicism in FMR1 full mutations. Front. Genet..

[3] Nolin S.L., Glicksman A., Ersalesi N., Dobkin C., Ted Brown W., Cao R., Blatt E., Sah S., Latham G.J., Hadd A.G. (2015). Fragile X full mutation expansions are inhibited by one or more AGG interruptions in premutation carriers. Genet. Med..

[4] Chen X., Wang J., Xie H., Zhou W., Wu Y., Wang J., Qin J., Guo J., Gu Q., Zhang X. (2015). Fragile X syndrome screening in Chinese children with unknown intellectual developmental disorder. BMC Pediatr..

[5] Kim E.Y., Page P., Dellefave-Castillo L.M., McNally E.M., Wyatt E.J. (2016). Direct reprogramming of urine-derived cells with inducible MyoD for modeling human muscle disease. Skeletal Muscle.

[6] Lazzeri E., Ronconi E., Angelotti M.L., Peired A., Mazzinghi B., Becherucci F., Conti S., Sansavini G., Sisti A., Ravaglia F. (2015). Human Urine-Derived Renal Progenitors for Personalized Modeling of Genetic Kidney Disorders. J. Am. Soc. Nephrol..

[7] Afzal M.Z., Strande J.L. (2015). Generation of Induced Pluripotent Stem Cells from Muscular Dystrophy Patients: Efficient Integration-free Reprogramming of Urine Derived Cells. J. Vis. Exp..

[8] Zhang S.-Z., Li H.-F., Ma L.-X., Qian W.-J., Wang Z.-F., Wu Z.-Y. (2015). Urine-derived induced pluripotent stem cells as a modeling tool for paroxysmal kinesigenic dyskinesia. Biol. Open.

[9] Zhang D., Wei G., Li P., Zhou X., Zhang Y. (2014). Urine-derived stem cells: A novel and versatile progenitor source for cell-based therapy and regenerative medicine. Genes Dis..

[10] Filipovic-Sadic S., Sah S., Chen L., Krosting J., Sekinger E., Zhang W., Hagerman P.J., Stenzel T.T., Hadd A.G., Latham G.J. (2010). A novel FMR1 PCR method for the routine detection of low abundance expanded alleles and full mutations in fragile X syndrome. Clin. Chem..

[11] Tassone F., Pan R., Amiri K., Taylor A.K., Hagerman P.J. (2008). A Rapid Polymerase Chain Reaction-Based Screening Method for Identification of All Expanded Alleles of the Fragile X (FMR1) Gene in Newborn and High-Risk Populations. J. Mol. Diagn..

[12] Tassone F., Hagerman R.J., Chamberlain W.D., Hagerman P.J. (2000). Transcription of the FMR1 gene in individuals with fragile X syndrome. Am. J. Med. Genet..

[13] Zhou T., Benda C., Dunzinger S., Huang Y., Ho J.C., Yang J., Wang Y., Zhang Y., Zhuang Q., Li Y. (2012). Generation of human induced pluripotent stem cells from urine samples. Nat. Protoc..

[14] Sauer V., Tchaikovskaya T., Wang X., Li Y., Zhang W., Tar K., Polgar Z., Ding J., Guha C., Fox I.J. (2016). Human Urinary Epithelial Cells as a Source of Engraftable Hepatocyte-Like Cells Using Stem Cell Technology. Cell Transpl..

[15] Loesch D.Z., Sherwell S., Kinsella G., Tassone F., Taylor A., Amor D., Sung S., Evans A. (2012). Fragile X-associated tremor/ataxia phenotype in a male carrier of unmethylated full mutation in the FMR1 gene. Clin. Genet..

[16] Tassone F., Hagerman R.J., Loesch D.Z., Lachiewicz A., Taylor A.K., Hagerman P.J. (2000). Fragile X males with unmethylated, full mutation trinucleotide repeat expansions have elevated levels ofFMR1 messenger RNA. Am. J. Med. Genet..

[17] Pretto D.I., Mendoza-Morales G., Lo J., Cao R., Hadd A., Latham G.J., Durbin-Johnson B., Hagerman R., Tassone F. (2014). CGG allele size somatic mosaicism and methylation inFMR1premutation alleles. J. Med. Genet..

[18] Hadd A.G., Filipovic-Sadic S., Zhou L., Williams A., Latham G.J., Berry-Kravis E., Hall D.A. (2016). A methylation PCR method determines FMR1 activation ratios and differentiates premutation allele mosaicism in carrier siblings. Clin. Epigenetics.

[19] Mailick M.R., Movaghar A., Hong J., Greenberg J.S., DaWalt L.S., Zhou L., Jackson J., Rathouz P.J., Baker M.W., Brilliant M. (2018). Health Profiles of Mosaic Versus Non-mosaic FMR1 Premutation Carrier Mothers of Children With Fragile X Syndrome. Front. Genet..

[20] Tassone F., Longshore J., Zunich J., Steinbach P., Salat U., Taylor A.K. (1999). Tissue-specific methylation differences in a fragile X premutation carrier. Clin. Genet..

[21] Genc B. (2000). Methylation mosaicism of 5′-(CGG)n-3’ repeats in fragile X, premutation and normal individuals. Nucleic Acids Res..

[22] Bonarrigo F.A., Russo S., Vizziello P., Menni F., Cogliati F., Giorgini V., Monti F., Milani D. (2014). Think About It. J. Child Neurol..

[23] Bharadwaj S., Liu G., Shi Y., Wu R., Yang B., He T., Fan Y., Lu X., Zhou X., Liu H. (2013). Multipotential differentiation of human urine-derived stem cells: Potential for therapeutic applications in urology. Stem Cells.

[24] Zhang S.-Z., Ma L.-X., Qian W.-J., Li H.-F., Wang Z.-F., Wang H.-X., Wu Z.-Y. (2016). Modeling Neurological Disease by Rapid Conversion of Human Urine Cells into Functional Neurons. Stem Cells Int..

[25] Wang L., Wang L., Huang W., Su H., Xue Y., Su Z., Liao B., Wang H., Bao X., Qin D. (2013). Generation of integration-free neural progenitor cells from cells in human urine. Nat. Methods.

[26] Cheng L., Hu W., Qiu B., Zhao J., Yu Y., Guan W., Wang M., Yang W., Pei G. (2014). Generation of neural progenitor cells by chemical cocktails and hypoxia. Cell Res..

[27] Bento G., Shafigullina A.K., Rizvanov A.A., Sardão V.A., Macedo M.P., Oliveira P.J. (2020). Urine-Derived Stem Cells: Applications in Regenerative and Predictive Medicine. Cells.

